# Myasthenia Gravis Masquerading as an Idiopathic Unilateral Facial Paralysis (Bell's Palsy)—A Very Rare and Unique Clinical Find

**DOI:** 10.3389/fneur.2020.00709

**Published:** 2020-07-28

**Authors:** Marwa Elnazeir, Siddharth Narayanan, Pradeepthi Badugu, Abid Hussain, Tamour Tareen, Alexi R. Hernandez, Wei Liu, Adriana E. Palade, Martin E. Brown

**Affiliations:** ^1^Department of Neurology, University of Louisville, Louisville, KY, United States; ^2^Department of Surgery, University of Louisville, Louisville, KY, United States; ^3^Department of Medicine, University of Louisville, Louisville, KY, United States

**Keywords:** autoimmune, acetylcholine, muscle weakness, facial palsy, stroke

## Abstract

Myasthenia gravis (MG) is an uncommon autoimmune neuromuscular junction disorder manifesting as fluctuating weakness of skeletal muscles. To add to its repertoire of mimicking a wide range of neurological disorders, the present case report is, to the best of our knowledge, the very first to describe MG masquerading as an idiopathic unilateral facial paralysis (Bell's palsy, BP). Our case report is distinct, highlights a novel clinical occurrence, offers new insights of how different neurological disorders may overlap with each other, and reminds neurologists to have a very broad and thorough comprehension for effective diagnoses and treatment plans. Several other conditions that produce facial nerve palsy identical to BP have also been discussed.

## Introduction

Myasthenia gravis (MG) is an autoimmune disease mediated by antibodies that bind to the acetylcholine receptors (AChRs) or to functionally related molecules associated with the postsynaptic membrane of the neuromuscular junction (1). Its incidence is ~20 per 100,000 in the USA (2), with the majority (~80%) of patients having antibodies against AChRs that cause receptor loss and postsynaptic membrane damage, possibly as a consequence of complement activation (3).

The classic presentation of MG is a fluctuating degree of weakness of ocular, orofacial, limb, or respiratory muscles that worsens with sustained activity and/or later in the day and improves with rest (4). MG may be either ocular (restricted to the ocular and extraocular musculature) or generalized (affecting other muscle groups) with a pattern of weakness which is sometimes markedly asymmetric, particularly the ocular symptoms (1).

Though classically characterized by fluctuating muscle weakness, MG is often called “the great imitator” as it may present as a persistent, focal weakness mimicking a wide range of neurological disorders including stroke. We present a unique and interesting case of a female patient initially transferred from an outside emergency department (ED) for a possible stroke, presented to our facility with signs and symptoms of unilateral cranial nerve 7 weakness, or Bell palsy (BP), and was eventually diagnosed with MG.

## Case

A 66-year-old right-handed Caucasian female with a past medical history of hypertension and depression was transferred to our facility for a possible stroke after presenting to an outside hospital ED with acute right facial droop and diplopia. She reported that the facial droop was new but the diplopia was not. Once in our facility, an extensive history was gathered by the neurology team, including questions about fatigue, dysphagia, ptosis, or any other focal or lateralized weakness; these symptoms were denied by the patient. She did admit to a generalized weakness but denied any diurnal worsening of this weakness or any of her other symptoms. The physical examination determined that she had a right upper and lower facial weakness and no active diplopia or ophthalmoparesis, even after sustained upward gaze. The computerized tomography (CT) angiogram of the head and neck along with the remainder of her stroke work-up were also unremarkable. A magnetic resonance imaging (MRI) of the brain was performed with and without contrast to evaluate for any brainstem lesions and was unrevealing. The levels of thyroid stimulating hormone and skeletal muscle enzymes were normal. She was diagnosed with right BP and started on acyclovir and steroids, and discharge orders were placed.

Before being discharged, she began to develop slurred speech, hoarseness, and an increase in her right facial weakness which was now accompanied by mild ptosis. She was admitted and underwent a bedside swallowing evaluation, which was abnormal. A modified barium swallow study was performed and suggested poor relaxation and coordination of the upper esophageal sphincter. The ear, nose, and throat (ENT) specialist evaluated the patient and recommended an esophageal dilation with Botox injection. A taste test to evaluate 7th cranial nerve function was performed at bedside and was normal. Concerns for other diagnoses were raised, including MG. The patient underwent a modified Tensilon test using 2 mg pyridostigmine administered intravenously with atropine at bedside for any bradycardia. About 30 s after pyridostigmine administration, the patient's ptosis and dysarthria completely resolved. Her dysphagia also improved, and she drank a few sips of water. All supportive evidence was thus in favor of the working diagnosis of MG. The ENT recommendation for botulinum toxin injections was denied once the Tensilon test was positive. The electrodiagnostic testing (repetitive nerve stimulation and single fiber electromyography) was deferred as a result of the positive Tensilon test and response to pyridostigmine, demonstrating a neuromuscular junction abnormality. Autoantibody lab testing was pending to further confirm MG diagnosis.

Plasma exchange (PLEX) was initiated for 5 days and the patient responded with a near-complete resolution of the right eye ptosis, facial droop, and dysphonia. She continued to fail the swallow evaluation initially but was able to pass for a puree diet after PLEX. A chest CT revealed no evidence of a thymoma or any residual thymic tissue. The patient was educated on aspiration precautions and discharged. Two weeks later, the acetylcholine receptor antibody levels (anti-AChRs) were found to be elevated, confirming the diagnosis of MG (AChR binding antibody: 5.11 [normal 0–0.24]; AChR blocking antibody: 66 [normal 0–25]; AChR modulating antibody: 44 [normal <21]).

## Discussion

A review of literature on MG presenting as other neurological disorders reveals no similar findings to ours despite several other published atypical MG findings (5, 6), making this report a distinctive clinical find. Based on the concerns raised by the outside hospital ED, our patient was initially evaluated for a possible stroke (7). Previous reports have shown MG as an uncommon stroke mimic, more specifically with unilateral facial droop (4, 8, 9). For our patient, the stroke work-up was unremarkable.

Idiopathic acute 7th cranial nerve palsy, or BP, occurs with a frequency of 20–30 cases per 100,000 people in the USA each year (7). Unlike the central causes of unilateral facial weakness, where only the lower half of the face is affected, the classical presentation of BP is an acute or subacute unilateral facial paralysis involving both the lower and the upper half of the face. This is often, but not always, accompanied by the loss of taste in the ipsilateral anterior two-thirds of the tongue. In this most common form of facial palsy, the lesion is in the periphery, affecting the facial nerve near the geniculate ganglion, as it passes through the facial canal. In rare cases, BP can result from a brainstem lesion, at the level of the ipsilateral facial nucleus or the facial nerve fibers before they emerge from the pons (10).

Many conditions can produce unilateral facial weakness, identical to BP. Structural lesions in the parotid gland or the inner ear such as salivary gland tumors or a cholesteatoma can produce cranial nerve seven compression and paralysis. Unilateral facial weakness may also occur as sequelae of Lyme disease, Ramsay Hunt syndrome, variants of Guillain–Barré syndrome (GBS), otitis media, sarcoidosis, and some influenza vaccines (11). While these conditions may present as isolated facial nerve palsies, they usually have additional features that distinguish them from BP and thus were excluded in the differential diagnosis of our patient.

Lesions of the central nervous system (CNS) such as from multiple sclerosis or stroke can also cause unilateral facial weakness. In contrast to peripheral lesions, however, supranuclear lesions affecting facial expression do not paralyze the forehead on the affected side, resulting in a unilateral facial paralysis that spares the forehead. Often, there is at least some weakness of extremities on the affected side as well. A brain MRI without abnormalities made a CNS etiology much less likely. Our patient presented with mild right eye ptosis, dysphagia, and dysphonia. Some reports suggest that as many as 50% of MG patients present with ocular symptoms of ptosis, diplopia, or both as the primary presentation of which 15% of patients also present with bulbar symptoms that include dysarthria, dysphagia, and fatigable chewing (12).

**Figure 1:**
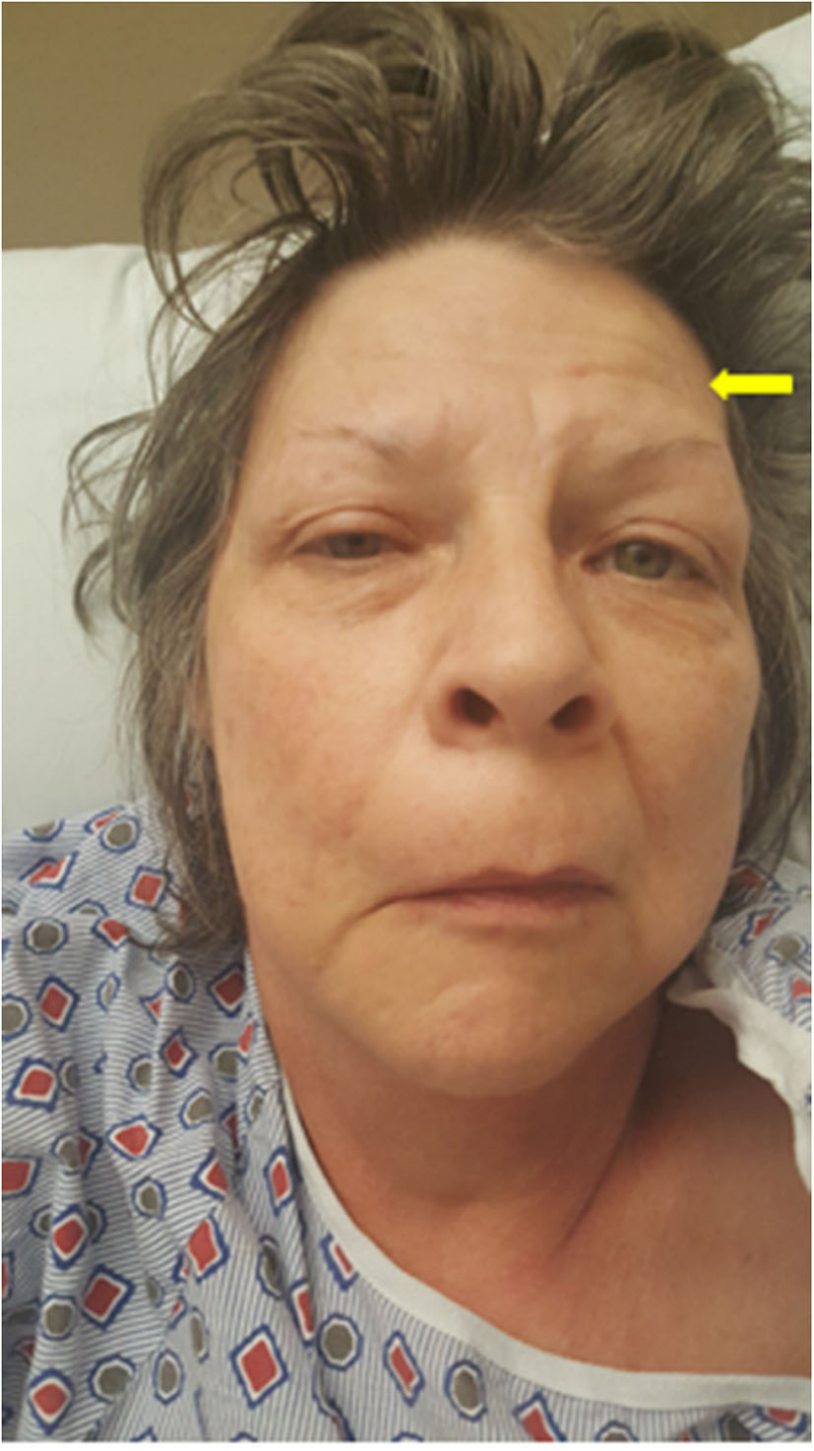
Myasthenia gravis masking as unilateral facial palsy. Image of the patient showing a right facial droop, mild right eye ptosis, and an unfurrowed right forehead. The yellow arrow points to the frontalis sign, suggesting that she is using her (partially weakened) frontalis muscle to overcome the ptosis.

In our patient, the lower motor neuron facial palsy was characterized by a unilateral paralysis of all muscles of facial expression for both voluntary and emotional responses. The forehead was unfurrowed (Figure 1). Viral infections have been associated with BP (11), hence our patient was initially started on a dose of acyclovir. However, despite this treatment, the patient developed slurred speech, had increased facial weakness, and failed a swallowing evaluation, all of which raised a suspicion for MG.

Oropharyngeal muscle weakness in MG patients produces dysphagia and dysarthria (12). When dysphagia is prominent, the patient may be unable to swallow medications or consume adequate food or liquids without aspiration. This inability to protect the airway is one form of a myasthenic crisis. In our case, bulbar involvement also raised the possibility of the pharyngeal–cervical–brachial variant of GBS (8), but this was deemed less likely based on the dramatic response to the modified Tensilon test. Structural brainstem lesions were also considered but were not detected by the brain MRI scan and would be expected to cause more than unilateral facial weakness. Lambert–Eaton myasthenic syndrome is not usually characterized by worsening fatigue over time and does not often produce dysarthria (8), and therefore, it was also not considered a likely diagnosis.

Therapeutic use of botulinum toxin type A (BoNT-A) was initially proposed by the ENT service for impaired relaxation of the upper esophageal sphincter. We declined this procedure as soon as the suspicion for MG was supported by the dramatic response to the modified Tensilon test. The diagnosis of MG was later confirmed by elevated levels of all three AChR antibodies. BoNT-A impairs the release of acetylcholine, which would further worsen neuromuscular transmission, hence patients with MG are exquisitely sensitive to BoNT-A injection owing to synergistic effects (13).

Finally, MG has recently been identified to have a stronger association with other autoimmune diseases of the nervous system than previously known. Thus, neurologists suspecting a new diagnosis of MG should bear in mind the possibility of co-existing autoimmune diseases of either the central or the peripheral nervous system (14, 15).

## Conclusions

MG can be aptly called a “neurologic chameleon.” Several reports identify MG as a mimic for a host of neurological disorders, thereby making it challenging for clinicians to quickly identify the correct diagnosis and proper treatment. A careful and detailed clinical history is a necessary step that can lead to a correct diagnosis of MG. Ours is the first case report, where MG mimics an idiopathic unilateral BP, after the patient was initially suspected of having a stroke. This report emphasizes the importance of having a thorough awareness and understanding of diverse neurologic conditions with overlapping features to facilitate accurate diagnosis, effective management, and avoid unnecessary and possibly harmful procedures and delayed treatment.

## Data Availability Statement

All datasets generated for this study are included in the article/Supplementary material.

## Ethics Statement

Written informed consent was obtained from the individual(s) for the publication of any potentially identifiable images or data included in this article.

## Consent for Publication

The patient consent was obtained and is part of the submitted documents.

## Author Contributions

ME collected the data. SN and ME analyzed the literature and wrote the manuscript. PB, AH, and TT helped analyze the literature. ARH, WL, AP, and MB reviewed and edited the manuscript. All authors read and agreed to the final version of the manuscript.

## Conflict of Interest

The authors declare that the research was conducted in the absence of any commercial or financial relationships that could be construed as a potential conflict of interest.
